# Coexistence of resistance to thyroid hormone and ectopic thyroid: ten-year follow-up

**DOI:** 10.1590/2359-3997000000214

**Published:** 2016-09-26

**Authors:** Man-Li Guo, Xiao Zheng, Liu-Xue Yang, Ya-Li Qiu, Liang Cheng, Shao-Gang Ma

**Affiliations:** 1 Department of Endocrinology and Metabolism Huai’an Hospital Affiliated to Xuzhou Medical College and Huai’an Second People’s Hospital Huai’an China Department of Endocrinology and Metabolism, Huai’an Hospital Affiliated to Xuzhou Medical College and Huai’an Second People’s Hospital, Huai’an, China; 2 Department of Endocrinology and Metabolism Second Hospital Affiliated to Guilin Medical College Guilin China Department of Endocrinology and Metabolism, the Second Hospital Affiliated to Guilin Medical College, Guilin, China; 3 Department of Neonatal Screening and Care Women and Children’s Hospital of Suqian Suqian China Department of Neonatal Screening and Care, Women and Children’s Hospital of Suqian, Suqian, China

## Abstract

Resistance to thyroid hormone (RTH) coexisting with ectopic thyroid is rare. Here we report a case of RTH with ectopic thyroid. A ten-year-old girl had been misdiagnosed as congenital hypothyroidism and treated with levothyroxine since she was born. Ten-year follow-up showed that the elevated thyrotropin was never suppressed by levothyroxine and no signs indicating hyperthyroidism or hypothyroidism despite elevated FT3 and FT4 levels. Therefore the girl developed no defects in physical and cognitive development. Pituitary adenoma was excluded by magnetic resonance imaging. Ultrasonography did not find the thyroid gland in the normal place, while the thyroid scan found a large lingual thyroid gland. The octreotide inhibition test showed a reduction in thyrotropin by 41.98%. No mutation was detected in the thyroid hormone receptor (THR) β, THRα, thyrotropin receptor (TSHR), and GNAS1 genes. To our knowledge, it is an interesting RTH case coexisting with lingual thyroid.

## INTRODUCTION

Resistance of thyroid hormone (RTH) is a rare genetic disease characterized by reduced tissue sensitivity to thyroid hormone. The hallmark of RTH is elevated circulating thyroid hormones with unsuppressed thyrotropin (TSH) (1). Most of RTH cases are caused by a mutation in the thyroid hormone receptor (*THR*) β gene (2). Recently, several reports described the RTH patients due to heterozygous truncating mutations in *THR*α (3). The clinical presentation of RTH is highly variable including hyperthyroidism, hypothyroidism and asymptomatic. Detection of RTH by a positive neonatal TSH screening test has been described in rare cases (4).

Ectopic thyroid is a rare embryological aberration. Together with thyroid agenesis and hypoplasia, thyroid ectopy is classified as thyroid dysgenesis. Whereas the mutations in the *NKX2-1, PAX8, FOXE1, NKX2-5, TSHR* genes have been reported in a minority of patients with thyroid dysgenesis (5). Lingual thyroid accounts for approximately 90% of ectopic thyroid tissue. However, the incidence is close to approximately 1:100,000. The majority of patients with lingual thyroid are asymptomatic (6,7). Subclinical and overt hypothyroidism can be observed in some patients, while hyperthyroidism is uncommon (8-10). The inactivating mutations in the guanine nucleotide binding subunit 1 gene (*GNAS1*) that encodes G protein α-subunit and causes mild TSH resistance in pseudohypoparathyroidism (PHP) type Ia is significantly rare (11).

The coexistence of RTH and ectopic thyroid is extremely rare and is difficult to distinguish, three cases have been reported (12-14). Here we describe an unusual patient with RTH and ectopic thyroid. We decided to screen the mutations in the *THR*α, *THRβ*,* TSHR*, and *GNAS1 *genes in the study. The patient in the present study was followed up for ten years.

## CASE REPORT

**Figure 1:**
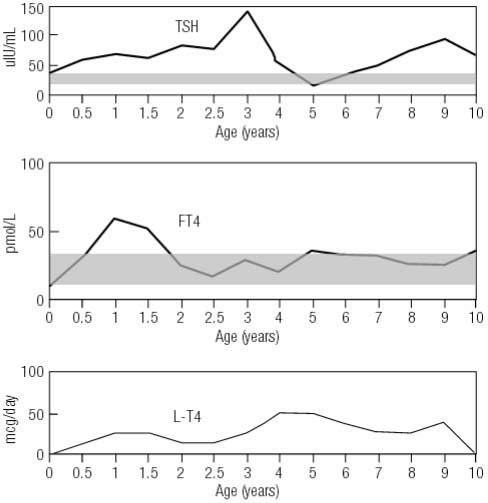
Clinical course of the levels of TSH and FT4 and doses of L-T4 during the 10 year follow-up.

The girl was born at 39 weeks in June, 2005. Newborn screening after birth revealed that the serum TSH level was 36.9 μIU/mL, while the FT4 was 9.01 pmol/mL (normal range: TSH, 0.5-5.0 μIU/mL; FT4, 8.56-25.6 pmol/mL). She had no signs of hypothyroidism, such as low growth rate, abdominal distention, mottled skin, open posterior fontanelle, prolonged jaundice, and lethargy. Thyroid ultrasound was not performed at that time, and the diagnosis of congenital hypothyroidism (CH) was retained. Levothyroxine (L-T4) replacement was given according to the misdiagnosis. However, the treatment of L-T4 had no effect on TSH levels, and FT4 were always elevated (Figure 1).

**Figure 2:**
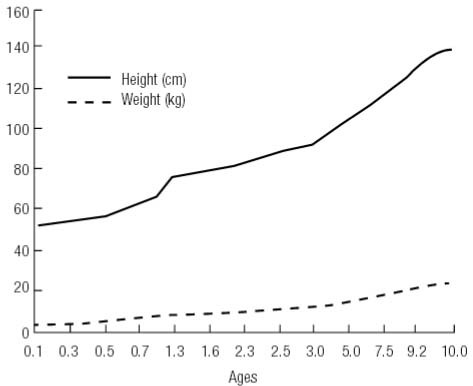
The growth rate (height and weight) of the patient during ten-year follow-up.

**Figure 3:**
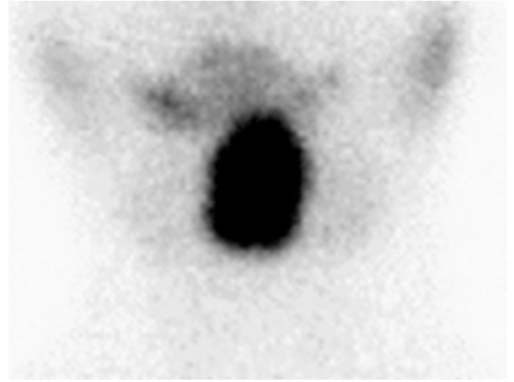
99mTc-pertechnetate thyroid scan showing a single enlarged lobe with intense uptake in the neck.

In August 2014, the patient was referred to our endocrine center due to abnormal thyroid function for 10 years. Physical examination showed that the patient had no symptoms of hypothyroidism or hyperthyroidism. Her weight was 25 kg, height was 132 cm, and heart rate was 81 beats/minute (Figure 2). She was slightly thin, but did not exhibit muscle wasting or tremor. We stopped the administration of L-T4 for 2 months and then confirmed the thyroid function of the patient and her family members (Table 1). The patient still showed elevated TSH levels despite high levels of FT3 and FT4, which suggests RTH. Ultrasonography did not find the thyroid gland in the normal place, while the thyroid scan found the enlarged lingual thyroid (Figure 3). Pituitary magnetic resonance imaging (MRI) showed no pituitary tumor in the sella turcica. Her bone age was normal and IQ was 90. Her parents’ thyroid function was normal without goiter. The octreotide inhibition test was negative, a decrease of up to 41.98% in TSH level was observed (Table 2). Interestingly, the level of platelet was 318-448 × 10^9^/L (normal range: 100-300 × 10^9^/L) during the 10 year follow-up.

**Table 1:** Detection of thyroid function of family members

Variables	Normal range	Daughter	Mother	Father
Age (years)	/	10	39	43
TSH (mIU/mL)	0.34-5.44	67.23	2.88	4.86
FT4 (pmol/L)	7.91-20.59	35.42	13.08	13.42
FT3 (pmol/L)	2.92-5.93	14.05	4.15	3.57
TT3 (nmol/mL)	1.30-3.10	5.89	1.89	1.52
TT4 (nmol/mL)	66.00-181.00	257.10	124.56	78.64
Tg (ng/ml)	1.15-130.77	329.60	66.74	37.87
TGAb (IU/mL)	0-34.00	< 10.00	-	-
TPOAb (IU/mL)	0-12.00	5.96	-	-
Thyroid volume	/	Enlarged	Normal	Normal

**Table 2:** Sandostatin inhibition test in the patient

Time	TSH (mIU/ml)	TT3 (nmol/l)	TT4 (nmol/l)	FT3 (pmol/l)	FT4 (pmol/l)	TSH/basic value (%)
0 h	59.050			12.22	34.14	100.00
2 h	46.330	3.26	188.90	10.86	30.47	78.46
4 h	45.130	3.30	186.20	11.11	31.87	76.42
6 h	28.320	3.29	190.00	12.46	33.00	47.96
8 h	27.700	3.12	180.81	11.73	32.35	46.91
24 h	24.790	3.40	191.04	12.76	34.01	41.98

Genomic DNA was extracted from peripheral blood leukocytes. Primers were designed to target the flanking intron regions of the exons. All exons of the *THR*β (MIM# 190160, GenBank NM_001128177.1),* THR*α (MIM# 199334.3, GenBank NM_190120)*, TSHR *(MIM# 603372, GenBank NM_000369.2) and *GNAS1* (MIM# 139320, GenBank NM_080425.3) genes were amplified using PCR. Sequence analysis indicated no mutation in the four genes.

## DISCUSSION

Here we describe a ten-year-old patient with RTH and lingual ectopic thyroid gland. Our patient had no mutation in the *THR*β,* THR*α,* TSHR *and *GNAS1* genes. The patient was misdiagnosed as CH due to the elevated level of TSH. Ten-year follow-up showed the serum levels of TSH, FT3, and FT4 were always high but without complications.

RTH is a rare syndrome characterized by decreased tissue responsiveness to thyroid hormone. RTH is mostly caused by mutations in the *THR*β gene, which encode for the thyroid hormone receptor beta unit (2). However, we did not find any mutations in the *THR*β,* THR*α*, TSHR *and *GNAS1* genes. According to previous reports, nearly 10% of the patients with RTH had no mutations in the coding region of *THR*β and 5% of the patients did not have mutations in both the *THR*β and *THR*α genes (3,15). There are multiple factors, including cofactors, transporters, deiodinases, and binding proteins, that may affect the actions of thyroid hormone (16). It is likely that defects in the factors that may affect the actions of thyroid hormone account for this patient’s phenotype.

The different degree of hypothyroidism can be observed in some patients with ectopic thyroid. The patient had the evaluated TSH but normal FT4 at birth. In our opinion, normal FT4 at birth could be related to ectopic thyroid. A similar situation can be observed in the previous literature (14).

Thyrotropin releasing hormone (TRH) stimulating and octreotide inhibition tests could be used in the diagnosis of RTH (17). However, TRH is no longer commercially available in China. The TSH value in our patient remained 41.98% after 24 hours of injection of somatostatin. In general, suppression of serum TSH in patients with TSH-secreting adenomas was significantly higher than patients with RTH. The elevated level of platelet was not reported previously, and the clinical significance needs more research.

LT-4 supplementation was necessary to the RTH patients with hypothyroidism (18-20). While other patients with RTH rarely require treatment, treatment is clearly necessary in the patient due to the high concentrations of TSH, which may cause further expansion of the lingual thyroid tissue (21). Bromocriptine (Brc), a dopamine agonist, has also been reported to suppress inappropriate TSH secretion in RTH, and it can be used alone or in combination with 3,5,3’-triiodothyroacetic acid (TRIAC) (22). However, the patient’s parents rejected L-T4 treatment for their child.

In conclusion, we describe the patient who met strict clinical and biochemical criteria for RTH but had no mutations in the considered genes. This patient was even more unusual because she had a lingual thyroid without hyperthyroidism or hypothyroidism symptoms.
